# Trends in operative treatment of the rectus diastasis A 13 year analysis of German nationwide hospital discharge data

**DOI:** 10.1007/s00423-025-03950-y

**Published:** 2025-12-22

**Authors:** C. Paasch, R. Lorenz, S. Lünse, M. Mainprize, O. Wendland, R. Hunger, R. Mantke

**Affiliations:** 1 Department for General and Visceral Surgery, University Hospital Brandenburg an der Havel, Brandenburg Medical School, Hochstraße 29, 14770 Brandenburg an der Havel, Germany; 2 Hernia Center 3+CHIRURGEN, Berlin, Germany; 3 Shouldice Hospital, Thornhill, Canada; 4 Faculty of Health Science Brandenburg, Brandenburg, Medical School, University Hospital Brandenburg/Havel, Brandenburg an der Havel, Germany

**Keywords:** Rectus diastasis, German hospital data, Longitudinal analysis, Real-world evidence

## Abstract

**Purpose:**

The indication for surgical treatment of rectus diastasis (RD) without a coexisting hernia remains controversial. Although guidelines exist, the lack of robust data allows only weak recommendations. This study aimed to provide comprehensive nationwide data on the surgical management of RD without hernia.

**Methods:**

This retrospective observational multicenter study analyzed anonymous data from the German nationwide hospital discharge dataset (2010–2023). Patients with coexisting hernia or under 18 years were excluded. The primary endpoint was the annual number of RD surgeries without hernia. Secondary endpoints included trends over 13 years, patient demographics, mesh use, and early postoperative complications.

**Results:**

A total of 2,768 cases were identified (mean age 46.2 ± 13.2 years; 76.2% female). The annual case number ranged from 120 to 253, with no consistent trend. A mesh was used in 28.0% (*n* = 775), while 72.0% underwent reconstruction without documented mesh. Data on surgical approach (open vs. minimally invasive) were not available. The overall early complication rate was 6.9%, with bleeding and wound infections most common. Male patients had significantly higher complication rates. Major limitations include potential coding bias, underreporting, and missing data on surgical technique.

**Conclusion:**

This is the first real-world big data analysis of RD repair without hernia in Germany. On average, 198 procedures are performed annually with a low complication rate. The findings support surgical treatment in selected symptomatic cases and emphasize the need for standardized coding and prospective registry data.

**Supplementary Information:**

The online version contains supplementary material available at 10.1007/s00423-025-03950-y.

## Introduction

Rectus Diastasis (RD) is an abnormality of the anterior abdominal wall characterized by a separation of the rectus abdominis muscles along the linea alba [1]. A thorough medical history and physical examination can diagnose most cases of RD. Symptoms include abdominal pain and discomfort, musculoskeletal and urogynecological issues, as well as negative body image and impaired quality of life [2]. In recent years, RD has been both defined and classified in the literature [3, 4].

According to the current guidelines of the European Hernia Society on the treatment of RD, surgery is generally indicated in all cases accompanied by symptomatic abdominal wall hernias [3]. However, due to the lack of robust data, the European guidelines only allow for weak recommendations. Ventral hernias with a defect size greater than 1 cm and concurrent RD should be repaired using mesh-based techniques [5]. A variety of surgical techniques, including innovative approaches, have been described in the literature for the repair of RD with concomitant ventral hernias; however, no comparative studies are available [6–10]. Aside from the recommendation to use mesh, no further guidance has been issued regarding which of the numerous surgical techniques may be advantageous for the repair of RD with or even without accompanying ventral hernias.

The indication for surgical treatment of RD in the absence of concomitant hernia remains controversial and is often considered a cosmetic procedure.

The aim of this study was to provide comprehensive nationwide data on the surgical treatment of RD in the absence of hernias.

## Methods

This is a retrospective multicentric analysis, conducted between January and March 2025 at the University Hospital Brandenburg an der Havel. The population-based diagnosis related group statistic (DRG-statistic) was queried by controlled remote data processing (Research Data Centre of the Federal Statistical Office and Statistical Offices of the Federal States, DOI: 10.21242/23141.2010.00.00.1.2.0—10.21242/23141.2023.00.00.1.2.0, own calculations). The dataset comprises all stationary hospital episodes in all hospitals within Germany. Hospitals and patients are anonymized, therefore neither institutes nor multiple hospital visits from individual patients can be identified. Ethical approval and patient consent were not needed for this study.

### Primary and secondary endpoints

The primary endpoint was the annual number of RD surgeries without a coexisting hernia. Secondary endpoints included 13-year trends in gender, age distribution, proportion of patients with mesh, type of used material, and early postoperative complications.

### Inclusion and exclusion criterias

**Fig. 1:**
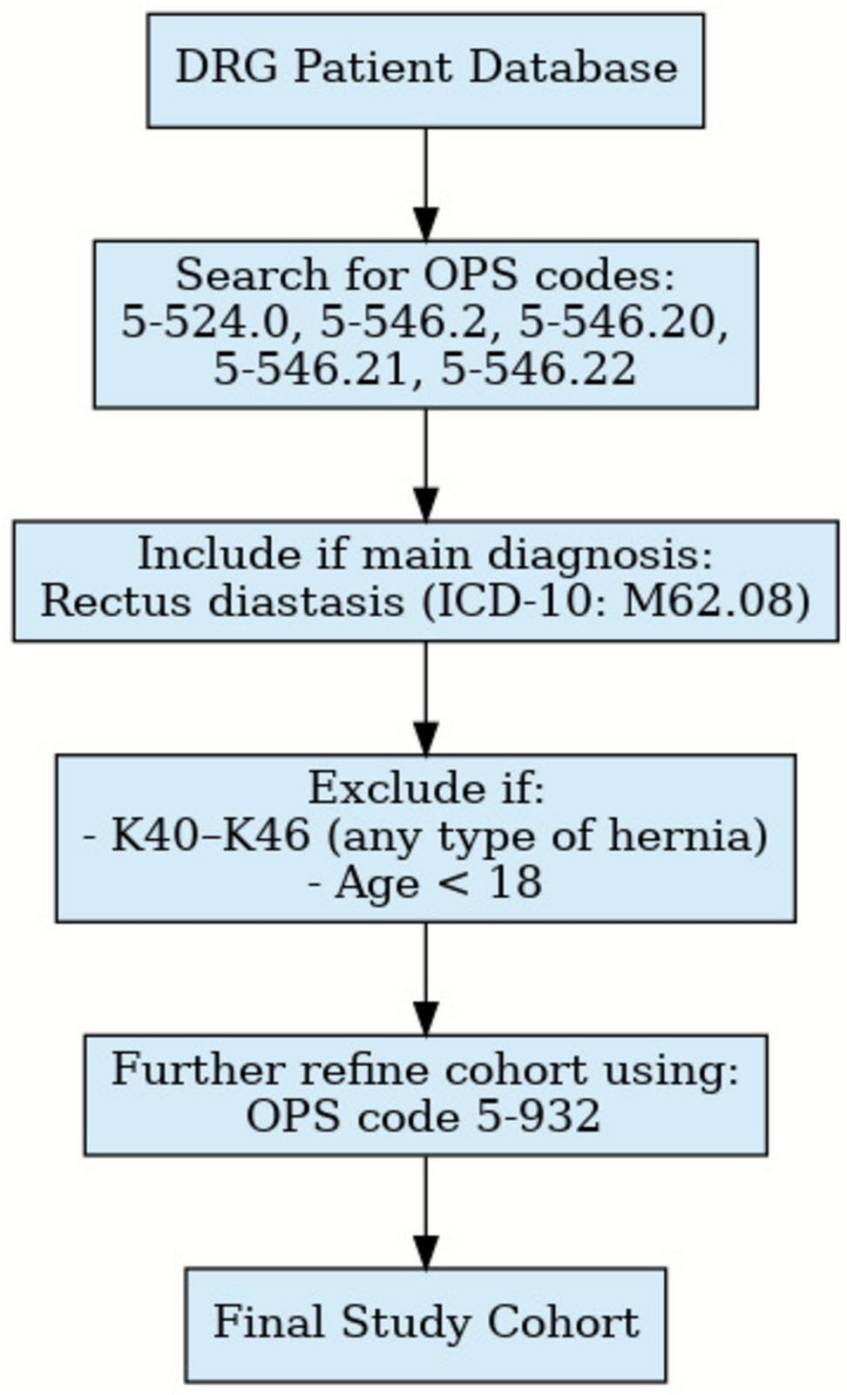
Flowchart on patients enrollement

To identify cases in which surgery took place for a RD (without a coexisting hernia) a search was performed for OPS (Surgical Operation and Procedure code classification system) and ICD-10-GM codes (according to the German modification of the International Classification of Diseases). Figure 1 depicts the detailed data flowchart with further information on exclusion and inclusion criteria. The data of patients over the age of 18 years with an ICD-10-GM code M62.08 (as main diagnosis) for a RD in combination with the OPS number 5–546.2 (Plastic reconstruction of the abdominal wall) and without the diagnosis of a coexisting hernia (ICD-10-GM: K40 – K46) were eligible for enrollment. OPS numbers for mesh placement were extracted, too (Table 1).

**Table 1:** Inclusion and exclusion criterias

Definition	Condition	Procedure/diagnosis codes
Inclusion procedures		
	Rectus diastasis as main diagnosis	M62.08
		5–524.0
	Plastic reconstruction of the abdominal wall	5–546.2, 5–546.20, 5–546.21,
		5–546-22. 5–546.2
Exclusion diagnosis		
	All types of a hernia	K40, K41, K42, K43
		K44, K45, K46

Age < 18

Sub-classifications of respective codes are included

### Study design

The DRG dataset includes all stationary and inpatient occurrences from all hospitals in Germany that take part in this enumeration system. A few hospitals (military hospitals) and cases (psychosomatic, psychiatric) are excluded from the system. The information on each inpatient episode is compulsorily collected by the individual hospitals and forwarded to the Institute for the Hospital Remuneration System. There, the data records are checked for plausibility, anonymized, and finally forwarded to the German Federal Statistical Office. This office uses controlled remote data processing and makes patients' information available for scientific research. The obligation to collect data, the criteria to be collected, the individual processing steps, and the possible uses are specified in detail in the law. At the patient level, the dataset includes demographic baseline information (gender, age), information on primary and secondary diagnoses (according to the ICD-10-GM) and medical procedures performed. The dataset further contains information on each hospital stay (type of admission, length-of-stay, and discharge status). Individual comorbidities were identified using the Quan-Elixhauser comorbidity definitions [11].

The dataset contains no information on Body Mass Index, ASA classification, and detailed surgical approach (open vs. minimally invasive).

### Statistical analysis

Medical procedures were identified using the appropriate OPS-codes. For analysis, especially presence of the OPS-codes 5–546.2 (plastic reconstruction of the abdominal wall) and 5–932 (type of material used for tissue replacement and tissue reinforcement) was identified. Particular attention was paid to considering the changes in OPS codes during the observation period. For example, the OPS code for the plastic reconstruction of the abdominal wall (5–546.2) did not contain any further differentiation options until 2017, when more detailed procedure subcodes were introduced. These subcodes allow the placement of mesh (onlay vs. sublay) to be identified. The procedure code 5–932 was even changed several times, with the coded materials being adapted in line with the change. In 2018 the code 5–932 was further extended and allowed the coding of the size of the material. Quantitative data were expressed as mean ± standard deviation (SD) and qualitative data as frequency and percentage. The primary endpoint was analyzed by estimating the slope and significance of a linear regression with year as predictor term and number of annual cases as outcome. In order to detect nonlinear temporal changes in the annual number of cases, a multivariate adaptive regression spline (MARS) analysis was performed [12]. Basically, it automatically splits the range of the predictor values into smaller subregions and fit separate linear regression models for each region. Model performance metrics for every possible split are determined and the model with the lowest prediction error, as assessed by cross-validation, is selected.

Whether a mesh was placed could be identified by the existence of the explicit code for its implantation (5–546.21 or 5–546.22) or the existence of the code 5–932, that coded the type of material used. Patient cohorts with and without complications were compared in a univariate analysis to identify possible risk factors. Therefore, depending on the type of the variable, chi-square tests (nominal variables), trend tests for proportions (ordered categorical variables), or t-Tests (continuous variables) were performed. Statistical analyses were conducted using R (version 4.2.3., The R Foundation, Vienna, Austria). All tests were two-sided and p-values < 0.05 were considered indicative of statistical significance.

## Results

A total of 2,768 cases were identified, with a mean age of 46.2 ± 13.2 years. The majority were female (76.2%). The duration of hospital stay was on average 5.39 (± 3.65) days. In 11.6% of cases an adipositas was coded. A total of 1,611 (58.2%) individuals had no comorbid conditions coded, and 220 (7.9%) patients had more than 2 comorbidities. A total of 5.7% (*n* = 159) were emergency surgeries.

### Primary endpoint

**Fig. 2:**
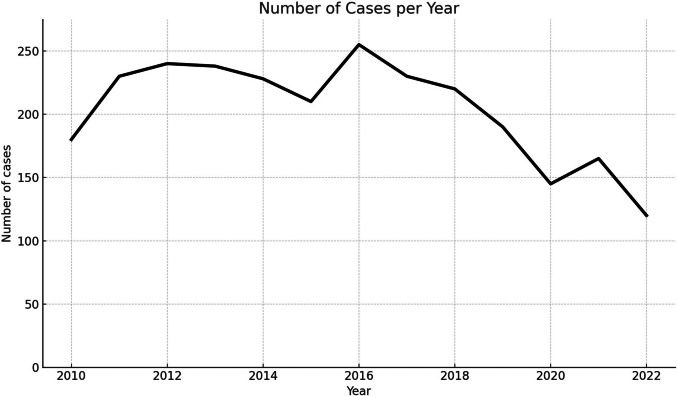
Annually case load of Rectus diastasis surgery in the abscence of a hernia from 2010 to 2023

An average of 197.7 (± 36.4) patients per year undergo surgery for RD without a hernia. The annual number of RD operations ranged from 120 in 2022 to 253 in 2016, with a significant trend over time (Fig. 2). The MARS model indicates an inflection point in the number of annual operations in the year 2016. In the early observation period, between 2010 and 2016, the annual number of cases slightly increased by 6.1 procedures per year (SE = 3.6, 95% CI: −3.1 to 15.4, *p* = 0.150). After 2016, the caseload declined by about 11.2 per year (SE = 5.1, 95% CI: −24.3 to 1.9, *p* = 0.079). Regarding the complete observation period, the number of cases declined by 5.5 cases per year (SE = 1.9, 95% CI: −1.2 to −9.7, *p* = 0.015).

### Secondary endpoints

Regarding the whole observation period from 2010 to 2023, an OPS procedure code for the type of implanted material was present in 775 cases (28.0%) indicating that a mesh was used. The majority of 1,993 patient (72.0%) underwent abdominal wall reconstruction without specification of mesh use. It must be noted that either no material was implanted or the used material was not coded by the hospital. A detailed analysis of the type of the material used showed that a partial-resorbable mesh was placed in 321 individuals (41.4%), a composite mesh in 54 (7.0%), and a non-resorbable mesh in 400 cases (51.6%).

After the introduction of the detailed OPS codes for implantation and positioning of the mesh in 2017, 1,226 patients were operated on. Of these operations, 503 (41.0%) had mesh placed whereas the remaining 723 patients (59.0%) had no mesh. When a mesh was implanted, in 163 cases (32.4%) the mesh was placed in an onlay and in 340 cases (67.6%) in a sublay position.

In summary, a mesh was used in 775 cases (28.0%), while the majority (*n* = 1,993; 72.0%) underwent abdominal wall reconstruction without the specification of mesh being used (Table S1).

The overall early postoperative complication rate was 6.9% (*n* = 191). The most common complication was bleeding, diagnosed in 107 patients (3.9%) and a postoperative wound infection in 85 patients (3.1%). The reoperation rate was 3.6% (*n* = 100). A total of 39 individuals (1.4%) were treated in an intensive care unit. A univariate analysis demonstrated that postoperative complications were more common in male than female patients (9.4% and 6.1%, respectively, *p* = 0.004) and persons with higher age (*p* < 0.001). In more recent years the rate of postoperative complications declined significantly (*p* = 0.003). While the complication rate was 9.0% between 2010 and 2016, it dropped to 5.5% in the period between 2017 and 2023.

Presence of early postoperative complications resulted in significantly (*p* < 0.001) prolonged length of hospital stay (average of 11.3 ± 8.5 days) compared to patients without any complication (5.0 ± 2.5 days).

## Discussion

In 2021, a guideline on the management of rectus diastasis (RD) was published in the British Journal of Surgery [3]. It addressed a total of nine key questions, of which only two — classification and definition — resulted in strong recommendations. Overall, the authors of this guideline emphasized the low quality of evidence and the weak strength of most recommendations. Consequently, this highlighted a strong need for further scientific data on this topic. For that reason, the study at hand was conducted. Key question 7 from the guideline states: “*What are the surgical treatment options in patients without concomitant hernias*?” A shared decision-making approach was generally recommended with both suture repair and mesh augmentation considered appropriate, but the quality of evidence was low and the strength of recommendation weak [3]. Against this background, it is not surprising that we revealed an annual rate of RD operations of only 197.7 (± 36.4). This may reflect the fact that most surgeons are aware of the low level of evidence and are therefore reluctant to move forward with surgery. There also seems to be a hesitation to use mesh, with only 769 (27.8%) cases reporting a mesh being implanted. This could be due to the fact that not only hernia surgeons operate on RDs but as cosmetic reasons can be an indication for RD, it also remains the domain of plastic surgeons [13]. However, the dataset does not allow identification of the surgical specialty or department involved in each case.

The safety of RD operations without concomitant hernias is of course an important issue. Overall, a postoperative complication rate of 6.9% was recorded. Most of them were wound infections and bleedings. Reoperation was performed in 3.9% (*n* = 107). In more recent years the rate of postoperative complications even declined significantly to 5.5%. Thus as an implication, the low and declining complication rate implies that rectus diastasis repair without concomitant hernia can be performed safely, which should encourage surgeons to consider surgical treatment in patients with clinically relevant symptoms. On the other hand, the present analysis, an analysis of discharge data, does not allow a detailed look at postoperative complications. It is not clear whether the reoperations were due to wound infection or haemorrhage.

Between 2010 and 2016, case numbers slightly increased, but after 2016 they declined significantly (–5.5 cases per year, *p* = 0.015). This decrease could reflect the 2017 coding modifications (new OPS subcodes), reimbursement changes, and a shift toward outpatient or privately financed procedures.

The data collected in our study comes from the whole of Germany, with no selection having taken place. For this reason, the results reflect the reality of RD surgery care in Germany. In the published guideline, central questions and results were developed based on clinical studies and reviews [3]. No discharge data records were used. In the following discussion we compared the findings of the only three relevant randomized controlled trials (RCTs) on RD surgery [14–16]. The question was: *Do these RCTs reflect reality of care?* An RCT by Schwedenhammer et al. (2019) mainly enrolled female patients (96.4%) with an average age of 43 years. RD surgery with or without mesh was performed in 57 people and there was no difference in quality of life or long-term pain between the two surgical groups [14]. In an RCT by Emanuelsson from 2016, 29 patients were assigned to retromuscular polypropylene mesh and 27 to double-row plication with Quill technology. The age ranged from 39.6 to 44.2 years, and the majority were female (97.7%), with no significant differences found in their results [15]. A Swedish RCT was conducted by Emanuelsson et al. in 2014. A total of 57 individuals were assigned to either a retromuscular polypropylene mesh or a double closure of the anterior rectus fascia using the self-closing Quill technology. No significant differences in outcomes were observed. Of the 64 participating patients, the majority (*n* = 62) were female and the average age at surgery was 40 years [16]. A comparison of these results with our results showed that the RCTs referenced reflect the reality of care with regard to age however, and for gender distribution. In our analysis, also more female patients were operated on for RDs without concomitant hernias (76.2%). In all RCTs on this topic, the vast majority of patients were also female (> 90%). The RCTs referenced here did not focus exclusively on RDs without concomitant hernias and therefore the populations may be naturally different. Furthermore, no RCT was conducted in Germany and for this reason, the question *Do these RCTs reflect reality of care in Germany?* could only be answered to a limited extent.

Male sex was associated with higher early postoperative complication rates. This could reflect the assumption that men undergoing RD surgery tend to have a higher comorbidity burden. It is conceivable that women are more likely to undergo RD surgery and in relative good or better health for cosmetic reasons and after completion of family planning. That could explain the lower rate of wound infection in this group. Unfortunately, the data set does not allow multivariate analyses to further investigate this observation. In addition, the ASA Score could not be extracted.

A total of 5.7% (*n* = 159) of the cases were emergency surgeries. One possible explanation is that a hernia was inadvertently not coded, and these cases may in fact have represented incarcerated hernias in the presence of a rectus diastasis (RD).

To our knowledge, the data set we collected is the one with the largest volume of RD surgeries without concomitant hernias. Nevertheless, the analysis and the discharge data set in general have limitations. Firstly, readmissions due to complications following RD surgery could not be extracted, nor could information on long-term outcomes, accurate ASA scores, or body mass indices. Therefore, no conclusions can be drawn regarding sex-related risk factors or risk-adjusted complication rates. Secondly, the accuracy of the data is highly dependent on the accuracy of individual surgeons in coding OPS and ICDs. Therefore, the present analysis should also be interpreted in the light of future registry data. In addition, they can only extracted after 2 years at the earliest, so no information on this topic is available for the years 2024 and 2025. The DRG dataset does not allow assessment of the surgical rationale for or against mesh use in non-hernia RD repair, which limits interpretation of the underlying decision-making process.

## Conclusion

This nationwide analysis provides the first real-world evidence on rectus diastasis (RD) repair without concomitant hernia in Germany, revealing that the procedure is performed infrequently and mostly without documented mesh use. The low and declining early complication rate implies that RD repair is a safe surgical option in selected patients, which may encourage surgeons to offer operative treatment in cases of clinically relevant symptoms. The observed sex differences in complication rates, variability in coding, and lack of mesh documentation underscore the need for standardized data collection and clearer guidelines. Moving forward, prospective multicenter studies and registry-based datasets are essential to define optimal indications, techniques, and patient selection in RD surgery.

## Supplementary Information

Below is the link to the electronic supplementary material.

Supplementary file1 (PDF 90 KB)

## Data Availability

The data are available based on reasonable request.
