# Supplementary material for: Trends in operative treatment of the rectus diastasis A 13 year analysis of German nationwide hospital discharge data
Source: Langenbecks Arch Surg. 2025 Dec 22;411(1):38. doi: 10.1007/s00423-025-03950-y (PMC12748285; doi:10.1007/s00423-025-03950-y)
Supplement: Supplementary file 1 — Supplementary file1 (PDF 90 KB) [file 423_2025_3950_MOESM1_ESM.pdf]

Category	Value
Total cases	2,768
Mean age (years)	46.2 ± 13.2
Female patients (%)	76.2%
Mean hospital stay (days)	5.39 ± 3.65
Adipositas coded (%)	11.6%
No comorbidity coded (%)	58.2%
More than 2 comorbidities (%)	7.9%
Mean annual RD surgeries (±SD)	197.7 ± 36.4 (120-253)
Overall change per year (2010–2023)	–5.5/year (**p=0.015**)
Mesh used (%)	775 (28.0%)
No mesh coded (%)	1,993 (72.0%)
Complication rate (%)	6.9% (n=191)
Bleeding (%)	3.9% (n=107)
Wound infection	3.1% (n=85)
Reoperation rate (%)	3.6% (n=100)
ICU treatment (%)	1.4% (n=39)
Complication rate male vs female	9.4% vs 6.1% (**p=0.004**)
Complication rate 2010–2016	9.0%
Complication rate 2017–2023	5.5%
LOS with complications (days)	11.3 ± 8.5

ICU Intermediate care unit; LOS Length of hospital stay; RD Rectus diastasis; SD Standard deviation
